# Preclinical efficacy of a gene therapy for *CHKB*-mediated muscular dystrophy

**DOI:** 10.1016/j.omta.2026.201766

**Published:** 2026-05-25

**Authors:** Mahtab Tavasoli, Mariam Alkandari, Gabriel Dorighello, Jennifer Devitt, Laura Hagerty, Jesse Damsker, Eric P. Hoffman, Christopher R. McMaster

**Affiliations:** 1 Department of Pharmacology, Dalhousie University, 5850 College St, Halifax, NS B3H 4H7, Canada; 2 Faculty of Medicine, Dalhousie University, 5850 College St, Halifax, NS B3H 4H7, Canada; 3 Reveragen BioPharma Inc, 155 Gibbs St, Rockville MD 20850, USA; 4 Binghamton University, State University of New York, 4400 Vestal Parkway East, Binghamton, NY 13902, USA

**Keywords:** dystrophy, myopathy, muscle, gene therapy, phospholipid, phosphatidylcholine, choline kinase, CHKB

## Abstract

Loss-of-function variants of the *CHKB* gene cause an autosomal recessive disease described as an early onset congenital megaconial (large peripheral mitochondria) muscular dystrophy. *CHKB* encodes choline kinase β, the first enzyme in the biochemical pathway for synthesis of the major membrane phospholipid phosphatidylcholine. *Chkb*^*-/-*^ mice recapitulate the human disease with affected skeletal muscle displaying a decrease in strength, myofiber atrophy, megaconial mitochondria, fat accumulation within muscle cells, and an increase in muscle injury. Here, we assessed the therapeutic potential of an AAV therapy for the treatment of *CHKB*-mediated muscular dystrophy. *Chkb*^*-/-*^ mice were injected once suborbitally with three different doses of recombinant AAV9 (rAAV9) encoding human *CHKB* under control of a constitutive and ubiquitous promoter (AAV9-*CHKB*). The AAV9-*CHKB*-treated mice were biochemically and phenotypically indistinguishable from the wild type mice. In the *Chkb*^*-/-*^ mouse model, all doses resulted in expression of the CHKB protein and restored choline kinase β enzyme activity, body and muscle weight, and normal muscle cell physiology, and they prevented lipid metabolism imbalance and increased the capacity to walk. These findings point to AAV9-mediated gene therapy as a potential treatment for *CHKB*-mediated disease.

## Graphical abstract

**Figure:**
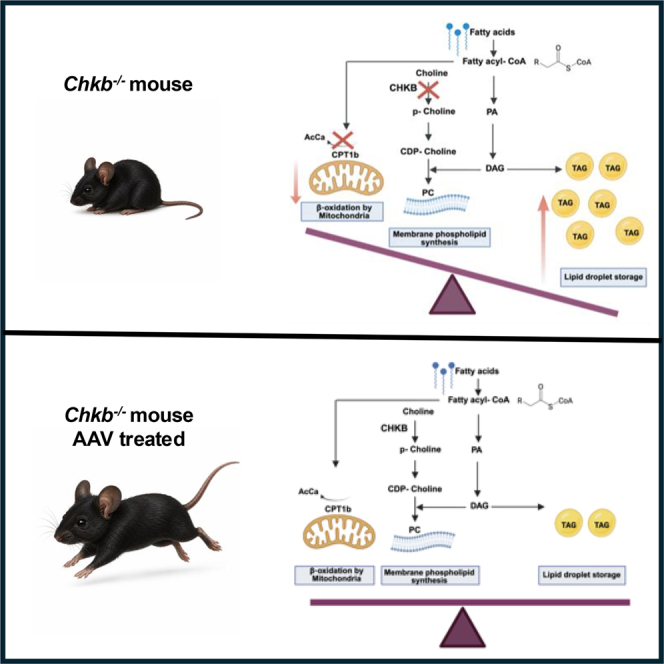

## Teaser

Congenital megaconial muscular dystrophy (MIM #602541) is an autosomal recessive disease caused by rare variants of the *CHKB* gene. McMaster and colleagues carried out a dose-ranging study of rAAV9 gene therapy delivery of the human *CHKB* gene to cells and to *Chkb*^-/-^ mice and determined its ability to ameliorate the disease.

## Introduction

Congenital megaconial muscular dystrophy (MIM #602541) is an autosomal recessive disease caused by rare variants of the *CHKB* gene^1^^,^^2^^,^^3^^,^^4^^,^^5^^,^^6^^,^^7^^,^^8^^,^^9^^,^^10^^,^^11^^,^^12^^,^^13^^,^^14^^,^^15^^,^^16^ located at chromosome 22q13.33. The spectrum of variations associated with this disorder includes missense, splice-site, stop/gain, and frameshift.^1^^,^^2^^,^^3^^,^^4^^,^^5^^,^^6^^,^^7^^,^^8^^,^^9^^,^^10^^,^^11^^,^^12^^,^^13^^,^^14^^,^^15^^,^^16^ The *CHKB* gene encodes the choline kinase β protein, a key enzyme in phospholipid biosynthesis.^2^^,^^10^^,^^12^ All cases of *CHKB*-mediated disease are due to a known (or presumed) loss of CHKB enzyme activity. *CHKB*-mediated muscular dystrophy is described as a progressive muscular dystrophy (100% of known patients) accompanied by intellectual disability and speech delay (96% of reported patients), with a subset of patients presenting with cardiomyopathy (30% of patients).^2^^,^^10^^,^^12^^,^^17^^,^^18^ The prevalence or incidence of *CHKB*-mediated muscular dystrophy is not known; to date, there have been less than 50 patients reported worldwide in the literature.^12^^,^^17^^,^^18^

Onset of disease is in infancy/early childhood (age ranging from 38 days to 16 years old, with most patients aged 2–4 years old), with the first phenotype most often being an increase in muscle weakness that can include hypotonia with head lag and a decrease in the ability to sit, stand, or walk.^2^^,^^3^^,^^6^^,^^9^^,^^10^^,^^12^^,^^14^^,^^16^^,^^19^ Earliest involvement is often in the posterior compartment as well as the anterior and medial compartments of the leg. In advanced disease, extensive fat accumulation in muscle can occur. Serum creatine kinase levels are mildly elevated and are 2- to 10-fold the normal levels.^3^^,^^12^^,^^13^ Muscle biopsy shows what has been described as myopathy with mild dystrophic changes as well as the megaconial phenotype of peripheral accumulation of enlarged mitochondria in muscle fibers.^2^^,^^3^^,^^10^^,^^12^^,^^15^^,^^19^^,^^20^^,^^21^ Brain MRI has not shown any obvious phenotypes despite reported intellectual disability. Patient survival varies from two years of age to early twenties with respiratory failure due to muscle weakness as the major cause of death, with some patients who present with cardiomyopathy dying from heart failure.^10^^,^^11^^,^^12^ There is no cure for *CHKB* disease, with management limited to physical and occupational therapy, speech therapy, and symptomatic interventions, which may include cardiac medications and/or cardiac pacemaker implantation, depending on the patient.

The *CHKB* gene encodes the 395 amino acid enzyme CHKB, a choline kinase that catalyzes the first step in the synthesis of phosphatidylcholine (PC) by the Kennedy pathway.^22^^,^^23^^,^^24^^,^^25^^,^^26^^,^^27^^,^^28^ Choline kinase is a soluble protein present in the cytoplasm, which produces phosphocholine from choline and ATP. Phosphocholine is subsequently converted to CDP-choline by the rate-determining step in the Kennedy pathway, cytidine triphosphate (CTP):phosphocholine cytidylyltransferase.^23^^,^^27^^,^^29^^,^^30^^,^^31^^,^^32^^,^^33^^,^^34^^,^^35^^,^^36^^,^^37^^,^^38^^,^^39^^,^^40^^,^^41^^,^^42^ The final step in the Kennedy pathway is a cholinephosphotransferase activity that converts CDP-choline and diacylglycerol to PC.^23^^,^^27^^,^^43^^,^^44^^,^^45^^,^^46^ PC is the most abundant phospholipid present in most eukaryotic cells comprising ∼50% of phospholipid mass. PC maintains membrane fluidity and bilayer formation and serves as a source for numerous second messengers.^27^^,^^32^^,^^39^^,^^41^

Study of the mouse model of *CHKB* disease has substantially increased knowledge of this disorder. *Chkb*^*-/*-^ mice develop a rostral-to-caudal muscular dystrophy, typically presenting within the first week after birth, and similar to the human counterpart, display a mild elevation in serum creatine kinase (2- to 3-fold).^24^^,^^28^^,^^47^^,^^48^^,^^49^^,^^50^ Skeletal muscles from *Chkb*^*-/*-^ mice show a decrease in strength measures with skeletal muscle histology observing myofiber atrophy, large peripheral mitochondria (megaconia), fat accumulation within muscle cells, and an increase in the level of muscle injury markers.^24^^,^^28^^,^^51^

While CHKB is a key component in PC synthesis, the level of PC in skeletal muscle of patients or mice models is not significantly different from the wild type (WT).^24^^,^^28^^,^^49^^,^^50^ Myofibers and other cells can obtain PC from dietary sources via the systemic circulation, and an increase in the capacity to import PC into muscle appears to normalize PC level.^24^^,^^49^^,^^50^ Molecular pathogenesis of the disease involves a toxic precursor model whereby affected muscle accumulates pathological levels of fatty acyl carnitines due to an inability to use diacylglycerol as a substrate downstream of the choline kinase step in the biochemical pathway for PC synthesis.^26^^,^^28^ Normally, fatty acyl carnitines are imported into mitochondria as a substrate for β-oxidation by Cpt1b, the first and rate-determining step in fatty acid β-oxidation.^20^^,^^26^^,^^28^ However, Cpt1b is downregulated in *Chkb*^-/-^ mice, with an inability to consume fatty acids by β-oxidation resulting in shunting of fatty acids into triacylglycerol, leading to a progressive increase in lipid droplets in muscle cells as the terminal lipid metabolic phenotype in affected muscle.^28^

As all cases of *CHKB*-mediated disease are due to loss of function of CHKB activity, gene replacement therapy presents as a possible therapeutic approach. We hypothesize that CHKB patients may be particularly responsive to gene therapy for the following reasons. *CHKB* should be amenable to the use of a ubiquitous promoter through gene therapy as its expression is not localized to a single cell or tissue type. CHKB is 45 kDa in size and its coding region is easily contained within rAAV vectors. The CHKB protein is soluble, present in the cytoplasm, and is not part of a protein complex where subunit stoichiometry can be important for complex assembly.^23^^,^^27^^,^^41^^,^^52^^,^^53^ Finally, the rate-determining step in PC synthesis is catalyzed by CTP:phosphocholine cytidylyltransferase, which lies downstream of CHKB in the pathway to synthesize PC, and thus, over-expression of the CHKB protein by gene therapy should does not affect the overall rate of PC synthesis.^29^^,^^30^^,^^31^^,^^32^^,^^33^^,^^34^^,^^35^^,^^36^^,^^37^^,^^38^^,^^39^^,^^40^^,^^42^^,^^52^

Recombinant AAV9 (rAAV9) has been shown to have tropism for skeletal muscle, heart, and CNS (key tissues in *CHKB* deficiency) and is known to be relatively non-pathogenic, non-integrating, and has extensively been used in clinical applications.^54^^,^^55^^,^^56^^,^^57^^,^^58^^,^^59^^,^^60^^,^^61^^,^^62^^,^^63^^,^^64^^,^^65^^,^^66^ In this current study, we carried out a dose-ranging study of rAAV9-mediated gene therapy delivery of the human *CHKB* gene to cells and to *Chkb*^-/-^ mice and determined its ability to ameliorate the disease.

## Results

### Plasmid and rAAV9-mediated *CHKB* expression restores choline kinase expression and enzymatic activity in cells

**Figure 1:**
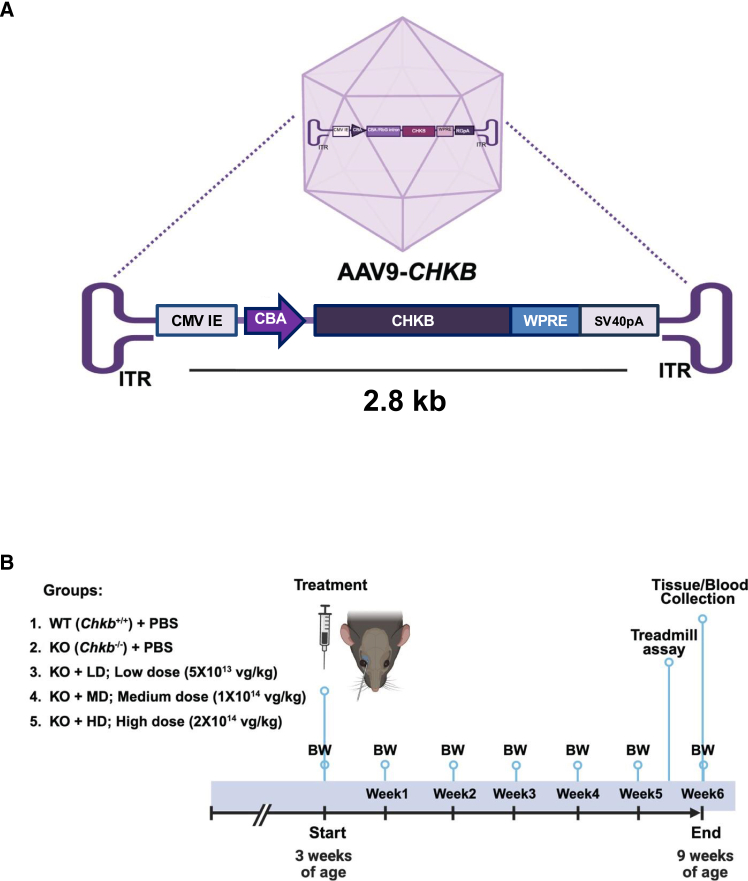
Experimental design for AAV9 -*CHKB* gene therapy (A) Experimental design of AAV9-*CHKB* treatment. The 5.3 single-stranded AAV9 vector carried human CHKB cDNA downstream of the chicken β-actin (CAG) promoter and followed by the WPRE sequence and SV40 polyA. (B) *Chkb*^+/+^ (WT) mice received PBS and served as healthy controls; *Chkb*^-/-^ (KO) mice received PBS or escalating doses of AAV9-*CHKB* (low dose (LD), 5 × 10^13^ vg/kg; medium dose, 1 × 10^14^ vg/kg; and high dose, 2 × 10^14^ vg/kg) at three weeks of age via a single RO injection (ROI). Body weight was measured weekly. At the end of the trial, a treadmill assay was performed (six weeks after AAV9-*CHKB* treatment), and blood and tissue samples were collected for analysis.

**Figure 2:**
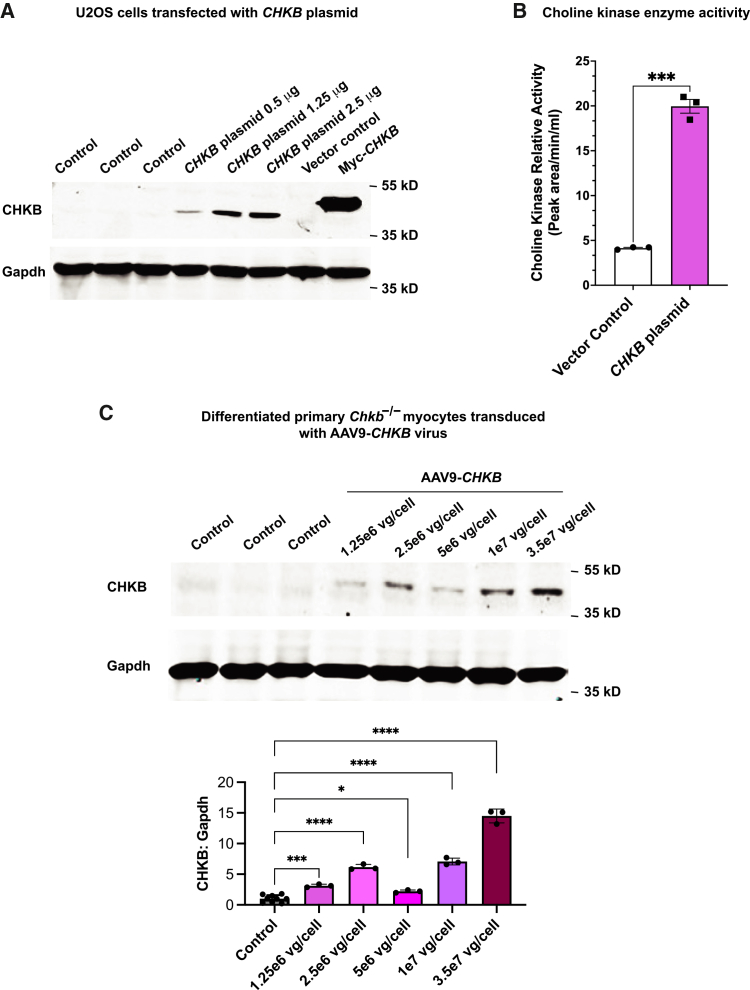
CHKB expression and enzymatic activity following *in vitro* plasmid or AAV9-*CHKB* delivery (A) Representative western blot showing CHKB protein expression in U2OS cells 72 h post-transfection with increasing doses of *CHKB* plasmid compared with controls. U2OS cells stably expressing Myc-CHKB or empty vector served as positive and negative controls, respectively. GAPDH was used as a loading control. (B) Choline kinase enzymatic activity assay was performed 72 h post-transfection on lysates from U2OS cells transfected with 1.25 μg CHKB expression plasmid. Data are presented as mean ± SD of three independent experiments. Statistical significance was determined using an unpaired *t* test; ∗∗∗*p* < 0.001. (C) Western blot of differentiated primary *Chkb*^-/-^ myocytes transduced with increasing doses of AAV9-*CHKB*. GAPDH served as a loading control. Quantification of protein expression was done by western blot. Data were analyzed by one-way ANOVA followed by Tukey’s multiple comparison test (*n* = 3). Data are presented as mean ± SD.

An rAAV9-based expression plasmid was constructed by placing the *CHKB* coding sequence downstream of the constitutive and ubiquitous cytomegalovirus (CMV)/chicken β-actin gene (CAG) promoter (Figures 1A and 1B). The plasmid was tested for CHKB protein expression via transfection into human U2O2 cells. A myc-tagged CHKB downstream of a CMV promoter was used as a positive control. Transfection of human U2O2 cells with increasing amounts of the rAAV9-based *CHKB* expression plasmid showed increasing levels of CHKB protein, as determined by western blot (Figure 2A). Assessment of CHKB choline kinase enzyme activity confirmed vector-driven production of functional CHKB enzyme, with U2OS cells transfected with *CHKB* expression plasmid showing a 5-fold increase in the level of the choline kinase product phosphocholine, indicating active choline kinase β enzyme activity (Figure 2B). The human *CHKB* expression plasmid was then packaged into rAAV9 (AAV9-*CHKB*) and tested for expresvsion in *Chkb*^*-/-*^ primary mouse myocytes. There is generally higher variability in transduction in primary cell lines for AAV9, and this variability tends to worsen when transduction efficiency is low, such as in muscle. We observed a dose-dependent trend in CHKB protein, as determined by western blot (Figure 2C).^67^ The results indicate that the *CHKB* expression plasmid produces active CHKB enzyme and that AAV9-*CHKB* can transduce *Chkb*^*-/-*^ mouse skeletal muscle cells *in vitro*.

### Systemic delivery of AAV9-*CHKB* increases CHKB expression and activity in skeletal muscle of *Chkb* knockout mice

*Chkb*^*-/-*^ mice were systemically treated with AAV9-*CHKB* by a single retro-orbital (RO) injection at three weeks of age. Three doses were tested: 5 × 10^13^ viral genomes per kg (vg/kg) (low dose, LD), 1 × 10^14^ vg/kg (medium dose, MD), and 2 × 10^14^ vg/kg (high dose, HD), along with saline placebo (Figure 1B). Mice were followed for six weeks subsequent to AAV9-*CHKB* treatment, at which point treadmill assays were performed and mice were sacrificed for histological and biochemical analysis.

**Figure 3:**
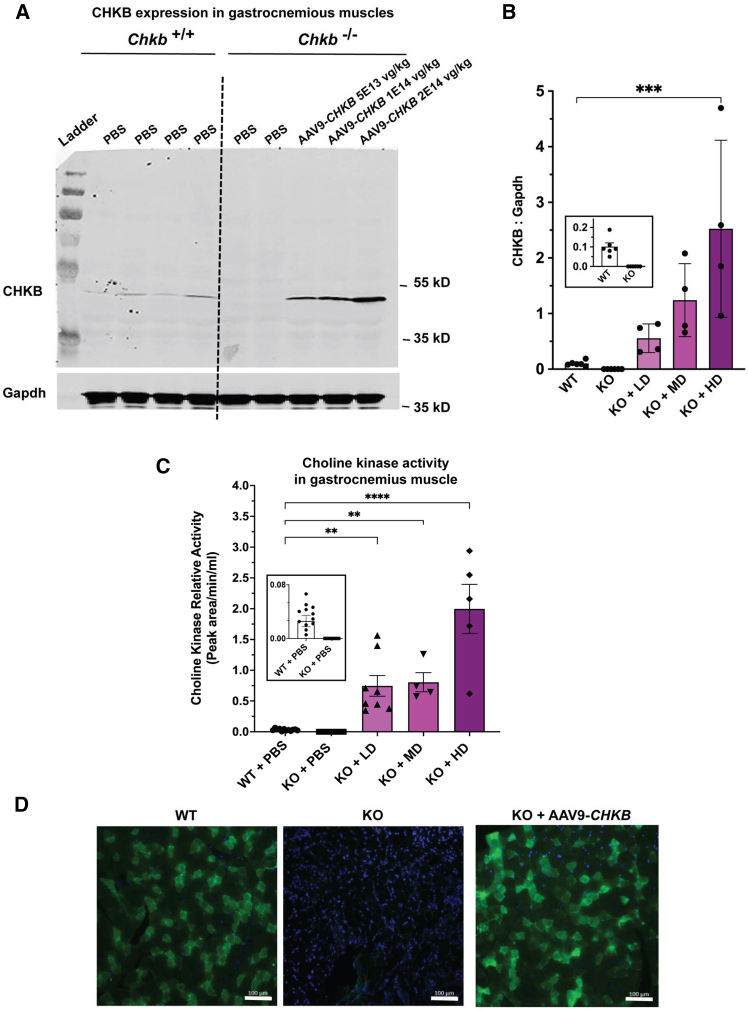
Restoration of CHKB expression and enzymatic activity in hindlimb muscle after systemic AAV9-*CHKB* treatment (A) Western blot analysis of gastrocnemius extracts from wild type (WT), KO (*Chkb*^-/-^), and KO mice treated with increasing doses of AAV9-*CHKB*: low dose (LD, 5 × 10^13^ vg/kg), medium dose (MD, 1 × 10^14^ vg/kg), and high dose (HD, 2 × 10^14^ vg/kg). GAPDH served as a loading control. (B) Quantification of CHKB protein normalized to GAPDH shows dose-dependent restoration. Inset shows zoomed in view of the WT and KO mouse levels to aid visualization. Data are shown as the mean ± SD of at least four biological replicates. Statistical significance was determined by one-way ANOVA followed by Tukey’s test for multiple comparisons (∗∗*p* < 0.01). (C) Muscle tissue lysates from WT, KO, and KO mice treated with low (LD), medium (MD), or high (HD) doses of AAV9-*CHKB* were prepared in enzyme activity buffer. Inset shows zoomed in view of the WT and KO mouse levels to aid visualization. Dots indicate individual mice; *n* = 9 (WT), *n* = 8 (KO), *n* = 6 (KO+LD), *n* = 4 (KO+MD), and *n* = 5 (KO+HD) mice per group. Inset shows zoomed in view of the WT and KO mouse protein levels to aid visualization. One-way ANOVA with Tukey’s multiple comparison test. Data are mean ± SD. ∗∗*p* < 0.01, ∗∗∗∗*p* < 0.0001. (D) Immunofluorescence staining of gastrocnemius sections for CHKB (green) and nuclei (DAPI, blue) demonstrates absence of CHKB in KO and robust recovery after AAV9-*CHKB* treatment. Representative of at least 3 individual mice per group; scale bars, 100 μm.

Western blot assay of human CHKB protein expression in the quadriceps muscle showed no detectable CHKB/Chkb protein in *Chkb*^*-/-*^ knockout (KO) mice, and a dose-dependent increase in CHKB expression upon treatment with AAV9-*CHKB* (Figure 3A). At 5 × 10^13^ vg/kg (low dose) treatment, the relative level of CHKB expressed in muscle was 6-fold higher than Chkb protein present in WT mice, with medium and high doses displaying 12- and 25-fold expression increases, respectively (Figure 3B). Choline kinase enzyme activity was not detectable in the *Chkb*^*-/-*^ gastrocnemius muscle, whereas AAV9-*CHKB* treatment showed an increase in choline kinase enzyme activity 30-, 33-, and 66-fold higher than WT levels at LD, MD, and HD, respectively (Figure 3C). Consistent with the CHKB western blot and enzyme expression assays, immunostaining of CHKB protein in quadriceps muscle showed the expected lack of CHKB/Chkb expression in *Chkb*^*-/-*^ mice. AAV9-*CHKB*-treated mice showed extensive CHKB protein expression, which localized to the cytoplasm with CHKB expressed at higher levels in the smaller diameter type 1 slow twitch oxidative myofibers (Figure 3D).

### AAV9-*CHKB* gene therapy corrects muscle histopathology

**Figure 4:**
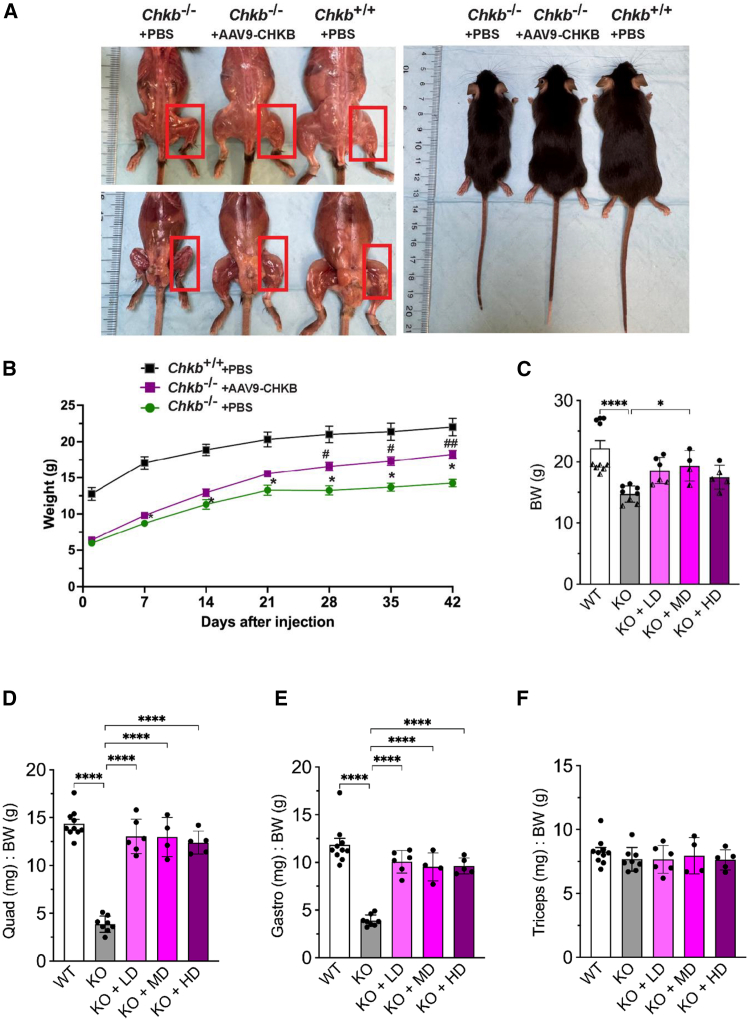
AAV9-*CHKB* therapy restores body weight and muscle mass in *Chkb*^*-/-*^ mice (A) Representative images of hindlimb and (B) whole-body morphology in WT, *Chkb*^-/-^, and AAV9-*CHKB*-treated *Chkb*^-/-^ mice. Treatment visibly restores body size and muscle mass in *Chkb*^-/-^ mice. (C) Weekly measurement of weekly body weight. (D) AAV9-*CHKB* treatment increases body weight gain compared with the untreated KO mice. Sample sizes: WT (*n* = 9), KO (*n* = 8), and KO + AAV9-*CHKB* (*n* = 15). Data are shown as mean ± SD. Statistical analysis was performed using one-way ANOVA with Tukey’s multiple comparisons test. ∗*p* < 0.05, KO + PBS vs. WT + PBS; #*p* < 0.05, ##*p* < 0.01, KO + AAV9-*CHKB* vs. KO + PBS. (E–G) mg/g body weight for quadriceps, gastrocnemius, and triceps at endpoint. Sample sizes: WT (*n* = 10), KO (*n* = 8), low-dose AAV9-*CHKB* (LD, 5 × 10^13^ vg/kg; *n* = 6), medium-dose (MD, 1 × 10^14^ vg/kg; *n* = 4), and high-dose (HD, 2 × 10^14^ vg/kg; *n* = 5). Data are presented as mean ± SD; dots represent individual animals. One-way ANOVA with Tukey’s multiple comparison test, ∗*p* < 0.05, ∗∗*p* < 0.01, ∗∗∗∗*p* < 0.0001.

Overt known phenotypes present in *Chkb*^*-/-*^ mice include a decrease in overall weight and decreased weight of affected muscle. Monitoring overall weight of *Chkb*^*-/-*^ mice treated with AAV9-*CHKB* against untreated mice determined that AAV9-*CHKB* significantly increased weight over time (Figures 4A–4D). The muscle phenotype in *Chkb*^*-/-*^ mice is rostral-to-caudal and as such, hindlimb muscles are known to be affected while forelimb muscles are less affected. This was confirmed in this study, with quadricep and gastrocnemius muscle weight decreasing to 30% and 32% of WT while triceps muscle mass was similar to WT. AAV9-*CHKB* treatment at all three doses tested increased muscle weight of quadricep and gastrocnemius to near WT level (Figures 4D–4F).

**Figure 5:**
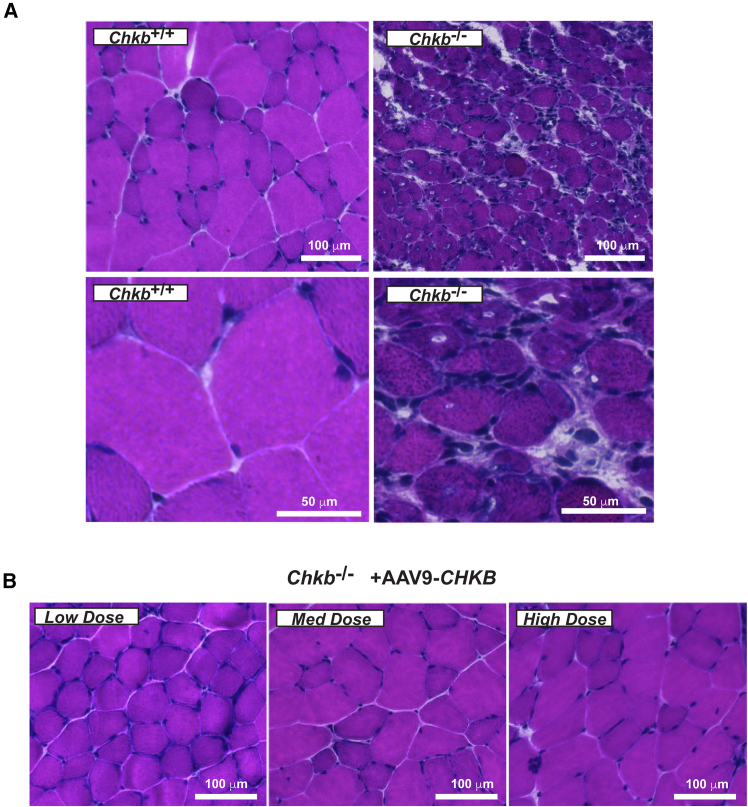
AAV9-*CHKB* treatment rescues muscle pathology Shown are (A) H+E stained cryosections from wild-type (WT) mice, *Chkb*^*-/-*^ sham-treated mice, and (B) AAV9-*CHKB*-treated *Chkb*^*-/-*^ mice. Severe muscle histopathology is seen in the sham-treated mice that is rescued by AAV9-*CHKB* treatment at all 3 doses (WT *n* = 10; *Chkb*^*-/-*^*n* = 8; and *Chkb*^*-/-*^ treated with AAV9-*CHKB* at low dose *n* = 6, med dose *n* = 4, and high dose *n* = 5).

Hematoxylin and eosin (H+E) staining of cryosections of cross-sections of the quadriceps muscles of WT and *Chkb*^*-/-*^ mice are shown (Figures 5A and 5B; Figure S1A). Wild-type muscle showed the typical eosinophilic myofibers with peripheral basophilic nuclei; some fiber size variation was due to smaller type I (slow twitch oxidative) and larger type II (fast twitch glycolytic) myofibers. The *Chkb*^*-/-*^ mice showed all myofibers to be markedly smaller and more variable in size, with greater basophilic (blue) staining of the cytoplasm consistent with mitochondrial proliferation. Increased cellular content of the endomysial connective tissue was also seen in the *Chkb*^*-/-*^ mice. In contrast, AAV9-*CHKB*-treated *Chkb*^*-/-*^ skeletal muscle showed rescue of all histopathology at all three doses (Figure 5B), with fiber size, staining patterns, and endomysial connective tissue histological phenotypes similar to WT muscle, and it also decreased the expression of muscle injury markers to WT levels (Figures S1B–S1D). Heterogeneity of CHKB expression was observed in *Chkb*^*-/-*^ skeletal muscle even though a ubiquitous promoter was used for *CHKB* expression in AAV9-*CHKB*, likely as it is known that there is differential myofiber-type transduction preference of AAV9 in mouse skeletal muscle (type 2x > 1 and 2a > 2 b) with resulting differences in protein expression despite the use of a ubiquitous promoter.^68^

**Figure 6:**
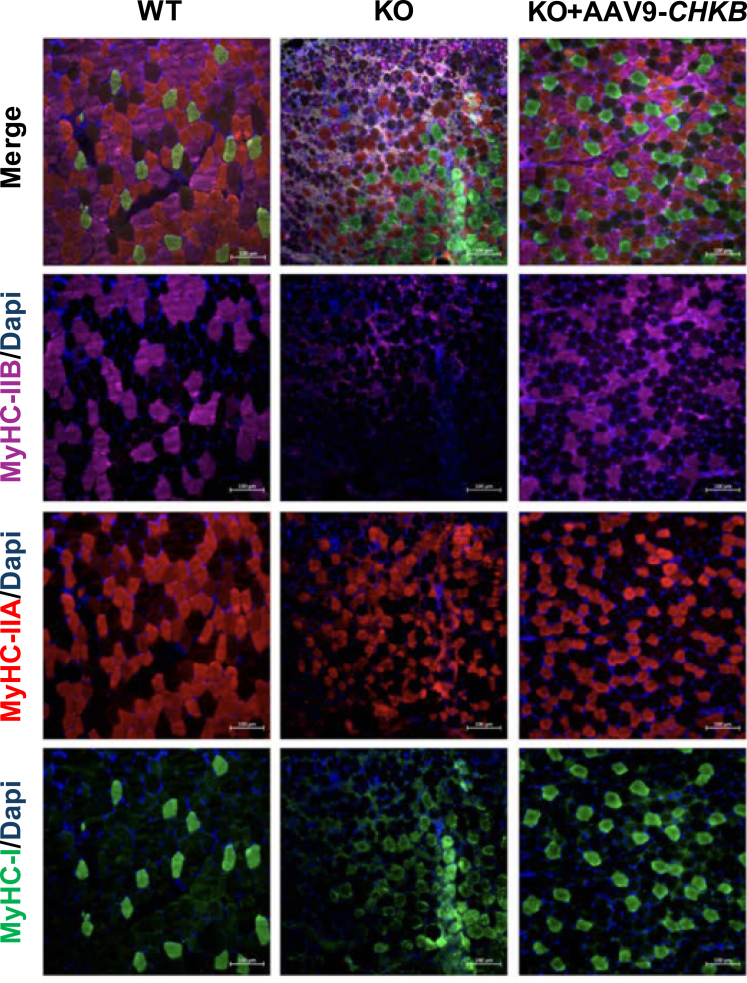
AAV9-*CHKB* treatment restores myofiber types Shown are single cryosections from gastrocnemius from wild-type (WT), *Chkb*^*-/-*^ (KO), and low dose AAV-*CHKB*-treated KO animals (similar results were observed for low, medium, and high doses). The sections were co-stained with DAPI (nuclei), and monoclonal antibodies for MyHC-I (type I, slow twitch oxidative), MyHC-IIA (type IIA, fast twitch oxidative-glycolytic), and MyHC-IIB (type IIB, fast twitch glycolytic) were used to identify myofiber types. WT muscle showed the expected pattern of a predominance of fast-twitch myofibers with full differentiation of fiber types. The *Chkb* KO muscle showed less well-differentiated myofiber types with all myofibers showing reduced myofiber size. These pathologies were largely rescued by treatment with AAV9-*CHKB* with full differentiation of myofiber types and increased myofiber diameter doses (WT *n* = 10*, Chkb*^*-/-*^*n* = 8; *Chkb*^*-/-*^ low dose *n* = 6, med dose *n* = 4, high dose *n* = 5).

Myofiber typing was done using monoclonal antibodies against myosin heavy chain isoforms (MyHC-I, type I slow twitch oxidative; MyHC-IIA, type IIA fast twitch oxidative-glycolytic; and MyHC-IIB, type IIB fast twitch glycolytic) labeled with distinct fluorophores in the same section (Figure 6) The WT gastrocnemius muscle showed the expected pattern of a predominance of fast twitch myofibers (both oxidative-glycolytic and glycolytic) and low numbers of slow twitch oxidative myofibers. The *Chkb* KO mouse showed poorer differentiation of fiber types, with all myofibers showing small diameter, as seen with H+E staining. Treatment of AAV9-*CHKB* rescued differentiation of fiber types, with increased diameter (myofiber size) toward WT.

### Aberrant mitochondrial phenotypes and lipid levels are corrected by AAV9-*CHKB* treatment of *Chkb*^*-/-*^ mice

**Figure 7:**
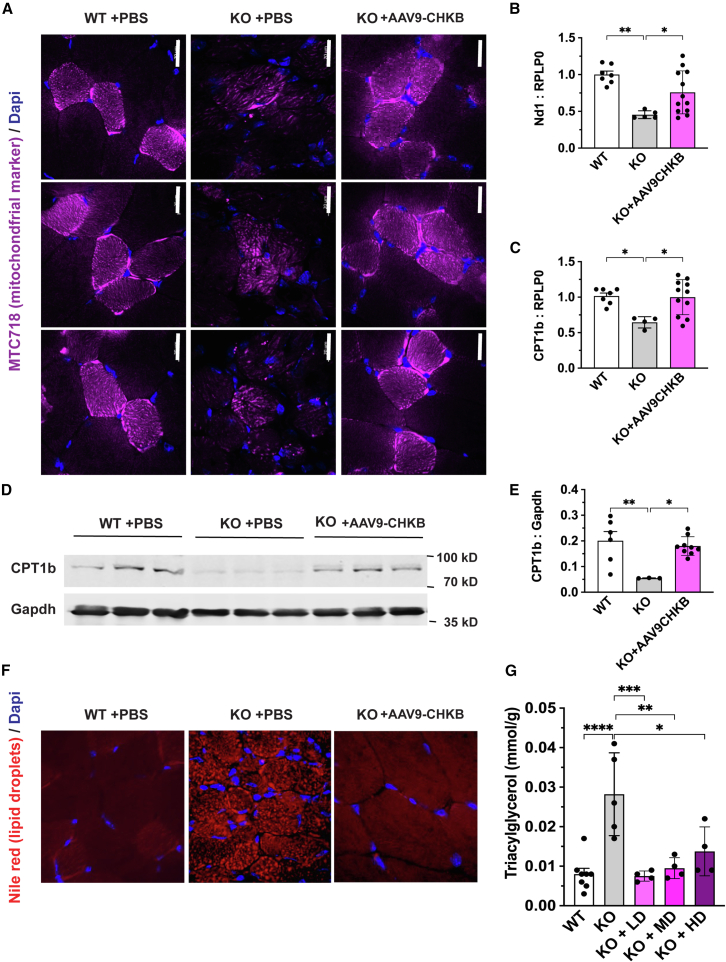
End stage defects in *Chkb*^*-*/-^ muscle are ameliorated by AAV9-*CHKB* (A) Immunofluorescence staining of muscle sections with mitochondrial marker MTCO1 (MTC718, magenta) and DAPI (blue); scale bars, 20 μm. (B) Relative gene expression of the mitochondrial gene *Nd1* normalized to Rplp0, determined by qPCR. (C) qPCR analysis of *Cpt1b* expression normalized to Rplp0. For (B) and (C), WT, *n* = 7; KO, *n* = 5; and KO + AAV9-*CHKB*, *n* = 12. One-way ANOVA with Tukey’s multiple comparison test was performed. Data are represented as mean ± SD; dots indicate individual mice. ∗*p* < 0.05, ∗∗*p* < 0.01. (D and E) Western blot of Cpt1b expression and its quantification in muscle extracts. GAPDH was used as a loading control. One-way ANOVA with Tukey’s multiple comparison test was performed. Data are mean ± SD of at least 4 replicates; dots indicate individual mice. ∗*p* < 0.05, ∗∗*p* < 0.01. (F) Nile red staining shows lipid droplet accumulation in *Chkb*^-/-^ gastrocnemius muscle that was reduced after AAV9-*CHKB*; scale bars, 50 μm. (G) Triacylglycerol (TAG) concentrations in gastrocnemius muscle were quantified. Data are mean ± SD for at least 4 mice per group; dots indicate individual mice. One-way ANOVA with Tukey’s multiple comparison test was performed. ∗*p* < 0.05, ∗∗*p* < 0.01, ∗∗∗*p* < 0.001, ∗∗∗∗*p* < 0.0001.

Mitochondria are a primary target in affected muscle of *CHKB* patients, and we had previously observed a decrease in the level of Cpt1b, the rate-determining step in mitochondrial β-oxidation. Immunofluorescence (IF) staining of WT myofibers with the mitochondrial marker MTC01 exhibited dense, fine, and well-organized mitochondrial distribution along the sarcoplasm (Figure 7A). In contrast, *Chkb*^-/-^ fibers showed enlarged mitochondria with markedly reduced staining density, leaving visible gaps within fibers, together with a disrupted and disorganized alignment. Following AAV9-*CHKB* treatment, mitochondrial morphology was restored toward WT, with recovery of density and size and re-establishment of a more continuous and organized pattern. Relative gene expression of the mitochondrial gene *Nd1* normalized to Rplp0 determined that *Chkb*^-/-^ mice exhibited a significant reduction in *Nd1* expression compared to WT (Figure 7B), consistent with impaired mitochondrial content. AAV9-*CHKB* treatment restored Nd1 transcript levels toward WT. Analysis of the level of the rate-determining enzyme for β-oxidation, *Cpt1b*, by qPCR suggested that it was significantly reduced in *Chkb*^-/-^ muscle compared with WT, (Figure 7C) and its expression was rescued by AAV9-*CHKB* treatment. Result of western blot of Cpt1b expression in muscle extracts was consistent with the qPCR result (Figures 7D and 7E), with AAV9-*CHKB* treatment increasing Cpt1b expression to WT level.

The end stage metabolic phenotype in affected muscle of *Chkb* KO mice is an accumulation of triacylglycerol stored as lipid droplets.^28^ Nile red staining for lipid droplets in gastrocnemius muscle resulted in a clear and obvious accumulation of lipid droplets in *Chkb*^*-/-*^ mice, while there was no obvious Nile red staining in WT mouse muscle (Figure 7F). Low, medium, and high dose treatment of *Chkb*^*-/-*^ mice with AAV9-*CHKB* resulted in the disappearance of lipid droplets in the gastrocnemius. Measurement of triacylglycerol level was consistent with this observation, with *Chkb*^*-/-*^ mice displaying a 3-fold increase in triacylglycerol in gastrocnemius muscle compared with WT, with all doses of AAV9-*CHKB* returning triacylglycerol level to WT (Figure 7G).

### Restoration of movement in *Chkb* knockout mice by AAV9-*CHKB*

**Figure 8:**
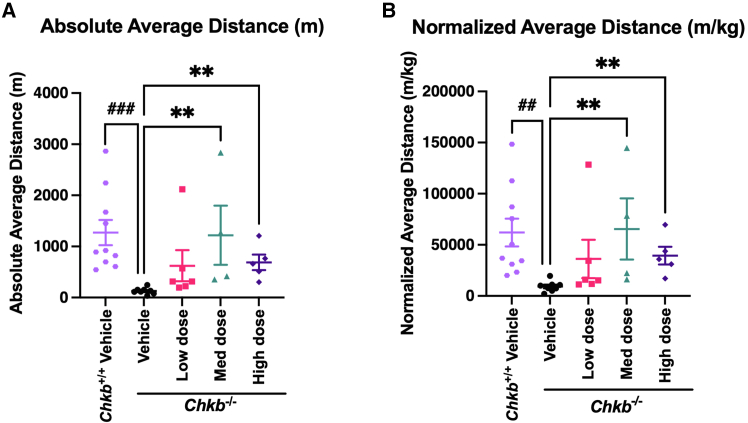
Functional improvements in locomotion and muscle strength following AAV9-*CHKB* treatment (A) Absolute average distance (m) and (B) normalized average distance (m/kg) run on the treadmill at 9 weeks of age (6 weeks post-injection). WT vehicle and *Chkb*^-/-^ vehicle groups were compared by unpaired *t* tests (###*p* < 0.001 for A; ##*p* = 0.003 for B). Non-parametric ANOVA (Kruskal-Wallis with Dunn’s post-hoc test) was used for multiple comparisons across treatment groups. ∗∗*p* < 0.005. Sample sizes: *n* = 10 (WT), *n* = 8 (KO vehicle), *n* = 6 (low dose AAV9-*CHKB*, 5 × 10^13^ vg/kg), *n* = 4 (medium dose AAV9-*CHKB*, 1 × 10^14^ vg/kg), and *n* = 5 (high dose AAV9-*CHKB*, 2 × 10^14^ vg/kg).

To determine whether the restoration of skeletal muscle phenotypes in *Chkb*^*-/-*^ mice treated with AAV9-*CHKB* resulted in an overt change in a known disability in these mice (walking/fatigue),^28^ the treadmill running assay was used. The *Chkb* KO mice were able to run a distance of 9,000 m/kg while WT mice ran 60,000 m/kg. Low, medium, and high doses of AAV9-*CHKB* increased the distance run by *Chkb*^*-/-*^ mice 3.8-, 7.5-, and 5.1-fold, respectively (Figures 8A and 8B).

### Reparation of heart injury by AAV9-*CHKB*

A known overt phenotype in 30% of *CHKB* patients, as well as in *Chkb* KO mice, is cardiomyopathy.^11^^,^^12^^,^^26^ Analysis of cardiac tissue of AAV9-*CHKB*-treated mice by western blot showed a dose-dependent increase in CHKB expression (Figures S2A and S2B). Cardiomyopathy was evident as previously reported as there was an increase in heart weight versus body weight in *Chkb*^*-/-*^ mice. Heart weight to body weight was normalized to WT upon treatment with all three doses of AAV9-*CHKB* (Figure S2C). H+E staining was similar between WT mice and *Chkb*^*-/-*^ mice as well as mice treated with all three doses of AAV9-*CHKB* (Figure S2D).

### No AAV9-*CHKB* treatment related adverse events were observed

Adeno-associated virus (AAV) use in the clinic can lead to adverse events and even death.^56^^,^^61^^,^^66^^,^^69^^,^^70^^,^^71^^,^^72^ No mouse deaths or obvious adverse events were observed in any of the AAV9-*CHKB*-treated mice. H+E staining of the liver determined that there were no obvious anomalies in either the WT or *Chkb*^*-/-*^ mice, and this was unaffected by AAV9-*CHKB* treatment as all groups showed preserved lobular architecture without evidence of necrosis, fibrosis, or fatty infiltration (Figure S3A). Liver mass, H+E staining, as well as the mass of other organs (Figures S3B and S4) was similar in WT and *Chkb*^*-/-*^ mice and did not substantially change upon AAV9-*CHKB* treatment. Analysis of serum alanine and aspartate transaminases (alanine aminotransferase [ALT] and aspartate aminotransferase [AST]) levels in *Chkb*^*-/-*^ mice at the end of the study determined that each was increased 2-fold compared to WT (Figures S3C and S3D). AST and ALT can be derived from damaged liver or muscle. To determine their potential origin, we assessed the change in the serum level of the Food and Drug Administration (FDA)-qualified liver specific damage marker glutamate dehydrogenase (GLDH) and observed that it also increased 2-fold in *Chkb*^*-/-*^ mice compared to WT (Figure S3E). The increase in serum AST, ALT, and GLDH levels was restored to normal by all three doses of AAV9-*CHKB* (Figures S3B–S3E).

## Discussion

This study demonstrates a significant therapeutic impact of a gene therapy through the expression of human *CHKB* in a mouse model of *CHKB* muscular dystrophy. This is the first ever example of a successful systemically administered gene therapy with enduring benefits in a preclinical model of this disease. The gene therapy developed uses rAAV9 and the ubiquitous CMV/CAG promoter with treatment at three weeks of age and evaluation of the efficacy of this gene therapy at nine weeks. We determined there was dose dependence of viral infection on recovery of expression of CHKB protein and choline kinase enzyme activity. All doses of AAV-*CHKB* tested restored body and muscle weight, recovered muscle cell pathology, restored lipid metabolism imbalance, increased the capacity to walk, and decreased muscle fatigue in the *Chkb*^*-/-*^ mouse model, with almost every parameter recovering to the point where it was near equivalent to WT mice.

Beyond the scientific rationale of rAAV9 tropism and the tissues affected in *CHKB*-mediated disease, our approach also seeks to partly leverage the extensive clinical safety experience of Zolgensma (over 4,000 patients treated in over 50 countries) and other gene therapies as we move toward a first in human trial.^55^^,^^56^^,^^58^^,^^59^^,^^61^^,^^66^^,^^69^^,^^73^^,^^74^^,^^75^^,^^76^^,^^77^^,^^78^^,^^79^^,^^80^ Zolgensma uses rAAV9 for the treatment of the neuromuscular disease spinal muscular atrophy (SMA) caused by recessive defects in the *SMN1* gene. The CMV/CAG promoter drives constitutive and ubiquitous *SMN1* expression in Zolgensma and drives *CHKB* expression in the rAAV9 used here. The recommended dose for Zolgensma is 1.1 × 10^14^ vg/kg, while in our preclinical mouse model of *CHKB*-mediated disease, a dose less than half, at 5 × 10^13^ vg/kg, was determined to be an effective treatment, which bodes well for prevention of potential adverse events and for its future development as a therapeutic. Future work could include further reduction in AAV-*CHKB* dose to determine the lowest effective dose for congenital *CHKB* disease treatment.

Disruption of the *CHKB* gene in patients is currently described as a muscular dystrophy in OMIM (MIM #602541), although early reports described *CHKB* disease being more similar to a congenital myopathy.^3^^,^^9^^,^^19^ Our current study of *Chkb*^*-/-*^ mice suggests the disease resembles a congenital myopathy more so than a muscular dystrophy. Muscular dystrophies generally result in muscle tissue loss and fibrous replacement of muscle, whereas congenital myopathies tend toward developmental defects of myofibers and muscle dysfunction.^17^^,^^18^^,^^55^^,^^57^^,^^81^^,^^82^^,^^83^^,^^84^^,^^85^^,^^86^^,^^87^ Our pathological analysis of affected muscle in *Chkb*^*-/-*^ mice determined that myofibers were present but were markedly smaller and more variable in size, with little fibro-fatty replacement. The fact that all muscle and biochemical parameters could be recovered by AAV-*CHKB* treatment also points toward full rescue of a biochemical and developmental defect, rather than muscle cell loss as the major muscle pathology in *Chkb*^*-/-*^ mice, consistent with congenital *CHKB* disease being more of a congenital myopathy than a muscular dystrophy. Future work to aid in answering this question and expanding the potential utility of AAV9-*CHKB* could include treatment of later stage disease in *Chkb*^*-/-*^ mice and assessing its capacity to reverse disease phenotypes.

The majority of *CHKB* patients reported to date also present with a large variation in intellectual disability and speech delay, although these are not life limiting for the disease, while mouse studies have pointed to altered bone mass.^2^^,^^3^^,^^6^^,^^9^^,^^10^^,^^12^^,^^15^^,^^16^^,^^47^^,^^88^ Brain MRI of *CHKB* patients has not determined any obvious changes consistent with these phenotypes. As delivery of rAAV9 can effectively cross the blood-brain barrier in humans if delivered early in life,^56^^,^^61^^,^^63^^,^^64^^,^^65^^,^^89^ further work on the *Chkb*^*-/-*^ mouse model of the disease should be the subject of future work with regard to brain dysfunction due to *CHKB/Chkb* loss and the potential for AAV9-*CHKB* to treat the neurological aspects of this disease.

## Materials and methods

### Quantification of AAV9-*CHKB* viral genomes stock by digital PCR

Viral samples were produced by PackGene Biotech (Houston, USA). The viral genome (vg) titer of the AAV9-*CHKB* stock was determined using the QIAcuity Digital PCR System (Qiagen) and the QIAcuity Probe PCR Kit. Samples were serially diluted (10^-3^–10^-8^) in nuclease-free water to obtain concentrations within the quantifiable range. Each 12 μL reaction contained 3 μL of 4× QIAcuity Probe PCR Master Mix, 0.8 μM of each primer, 0.4 μM hydrolysis probe, and 4 μL of diluted viral stock. Two independent TaqMan probe assays targeting distinct regions of the *CHKB* expression cassette (the *CHKB* coding region and the woodchuck hepatitis virus posttranscriptional regulatory element (WPRE) element, referred to as set 1 and set 2) were used to verify assay concordance. Reactions were prepared in PCR tubes, mixed thoroughly, and transferred to a QIAcuity Nanoplate 8.5 K (low-volume, 12 μL/well). The sealed plate was run using the following cycling program: 95°C for 10 min (to ensure heat lysis of viral capsids), followed by 40 cycles of 95°C for 15 s, and 60°C for 30 s. Fluorescence was detected in the green channel only. Data were analyzed using QIAcuity Software Suite (Qiagen) with automatic thresholding. Viral genome concentration (copies/μL reaction) was multiplied by the appropriate dilution factor and expressed as viral genomes per mL (vg/mL) of the original stock. The final titer was reported as the mean ± SD of replicate wells from both assays, which agreed within 0.2 log_10_. Non-template controls were negative in all runs.

### U2OS cell transfection

U2OS cells (ATCC HTB-96) were maintained in Dulbecco’s Modified Eagle Medium (DMEM; Gibco) supplemented with 10% fetal bovine serum (FBS; Gibco) and 1% antibiotic-antimycotic (Gibco). For transfection, cells were seeded at a density of 0.6 × 10^6^ cells per well in 6-well plates to achieve approximately 70%–80% confluency on the following day, at which point transfection was performed. Cells were transfected with the CHKB expression plasmid AAV-*CHKB* using Lipofectamine 2000 (Thermo Fisher Scientific) according to the manufacturer’s protocol. Briefly, 0.5, 1.25, or 2.5 μg of plasmid DNA was diluted in 150 μL of Opti-MEM (Gibco) and mixed with 9 μL of Lipofectamine 2000 diluted in a separate 150 μL of Opti-MEM. The DNA-lipid complexes were incubated for 15–20 min at room temperature before being added to the cells. The following day, the medium was replaced with fresh growth medium. Cells were harvested 48 h post-transfection using 200 μL of RIPA lysis buffer for protein extraction. Total protein concentration was determined using a colorimetric assay (e.g., bicinchoninic acid [BCA] Protein Assay; Thermo Fisher Scientific), and 6 μg of protein in 30 μL of loading buffer was resolved per lane for immunoblot analysis of CHKB expression. U2OS cells stably expressing Myc-tagged CHKB and cells transfected with the corresponding empty vector served as positive and negative controls, respectively, as previously described.^28^^,^^51^

### Myocyte differentiation and viral transduction

Primary mouse *Chkb*^*-/-*^ myoblasts were cultured and maintained as previously described.^28^^,^^51^ For differentiation, one T75 flask at approximately 80% confluency was trypsinized and seeded into Matrigel-coated 48-well plates (Matrigel diluted 1:100 in phosphate-buffered saline [PBS]) at a seeding ratio of 1:28, with a final volume of 350 μL per well. Cells were allowed to reach 70%–80% confluency and then switched to differentiation medium (DMEM supplemented with 5% horse serum, 350 μL per well). Differentiation was carried out for 4 days, with the medium replaced every other day. For viral transduction, differentiated myocytes were incubated with AAV9-*CHKB* at final volumes of 1.25, 2.5, 10, or 35 μL of viral stock (5 × 10^13^ vg/mL) in 350 μL of differentiation medium per well. Cells were harvested 72 h post-transduction using 50 μL of RIPA lysis buffer per well for protein extraction and subsequent analysis of CHKB expression. The total protein yield from each well was used for immunoblot analysis.

### Myofiber typing

Skeletal muscle was frozen on cork platforms using liquid-nitrogen-cooled isopentane. Sections were cut to 10 μM and allowed to briefly dry. A PAP pen was used to create a hydrophobic barrier around the sections. Sections were blocked with mouse on mouse (Vector; MKB-2213) for 1 h at room temp and incubated in PBS with a 1:100 dilution of monoclonal antibodies obtained from the developmental studies hybridoma bank (DSHB) against myosin heavy chain type I (BA-D5), myosin heavy chain type IIA (SC-71), and myosin heavy chain type IIB (BF-F3) for 12 h at 4°C. Samples were washed with PBS and incubated in 1% BSA/PBS at 1:500 dilution of secondary antibodies from Jackson ImmunoResearch IgG2b-Alexa405, IgG1-Alexa488, and IgG-Alexa647 (catalog numbers #115-475-207, #115-545-205, and #115-585-075) for 2 h at room temp, washed with PBS, and mounted using Prolong Gold antifade.

### Animals and study design

All animal procedures were approved by the Dalhousie University Committee on Laboratory Animals and conducted in accordance with the Canadian Council on Animal Care (CCAC) guidelines. Animal husbandry conditions and the generation of *Chkb* mutant mice on the C57BL/6 J background have been previously described.^28^ Briefly, mice were housed in ventilated cages under standard conditions on a 13:11 h light-dark cycle. Male *Chkb*^+/-^ mice on the C57BL/6 J background were crossed with female *Chkb*^+/-^ mice of the same background to generate *Chkb*^+/+^ (WT), *Chkb*^-/-^ (KO), and *Chkb*^+/-^ littermates. The *Chkb*^-/-^ mutation is a 1.6 kb genomic deletion between exon 3 and intron 9 that produces a truncated mRNA and results in absence of Chkb protein expression.^24^^,^^28^ A total of 10 WT mice, 8 KO mice, 6 KO + low-dose (LD) mice, 4 KO + medium-dose (MD) mice, and 5 KO + high-dose (HD) mice were included in the study. *Chkb*^*-/-*^ mice were randomly assigned to treatment (high, medium, or low doses) or control groups. Gene replacement therapy was administered via RO injection of AAV9-*CHKB* viral particles carrying the human *CHKB* coding sequence under the CAG (CMV early enhancer/chicken β-actin) promoter. The recombinant vector genome contained the *CHKB* cDNA, WPRE, and SV40 polyadenylation signal, flanked by AAV2 inverted terminal repeats (ITRs). The AAV9-*CHKB* vector was delivered at doses of 2 × 10^14^ vg/kg (high), 1 × 10^14^ vg/kg (medium), or 5 × 10^13^ vg/kg (low) in a total injection volume of 30 μL per animal. Control mice (WT and *Chkb*^*-/-*^) received an equivalent volume of vehicle (PBS). Injections were administered between 19 and 24 days of age. Animals were monitored regularly for general health, body weight, and signs of adverse effects throughout the study. Between 51 and 63 days of age, mice underwent *in vivo* assessment of fatigability using a treadmill assay, as previously described.^28^ Mice were sacrificed between 65 and 69 days of age. Blood was collected, and tissues were either flash-frozen for subsequent analysis or frozen in isopentane for histopathology and IF staining.

### Mouse genotyping

Genomic DNA was extracted from ear punch biopsies using the AccuStart II Mouse Genotyping Kit (Beverly, MA, USA), following the manufacturer’s protocol. A single PCR amplification program was employed to simultaneously detect both the WT *Chkb* allele (amplified between exons 5 and 9) and the mutant *Chkb* allele (amplified between exons 2 and 10). Primers were synthesized by Integrated DNA Technologies (Coralville, IA, USA). The primer sequences used for WT genotyping were forward 5′-GTG GGT GGC ACT GGC ATT TAT-3′ and reverse 5′-GTT TCT TCT GTT CCT CTT CGG AGA-3′, yielding a 753 bp amplicon. For mutant genotyping, the primers used were forward 5′-TAC CCA CGT ACC TCT GGC TTT T-3′ and reverse 5′-GCT TTC CTG GAG GAC GTG AC-3′, yielding a 486 bp amplicon. For each mouse, a single PCR reaction containing both primer sets was performed. Samples producing two amplification bands were identified as heterozygous (*Chkb*^+/-^), while those yielding a single 753 bp or 486 bp band were classified as wild-type (*Chkb*^+/+^) or homozygous mutant (*Chkb*^-/-^), respectively.

### *In vivo* fatigability measurements

Mice were subjected to an enforced running protocol to evaluate fatigue resistance. The assay was conducted between 59 and 63 days of age. Each mouse was first acclimated to the treadmill, then run on a horizontal belt for 5 min at 5 m/min, after which the speed was increased by 1 m/min every min, as previously described.^28^ The total distance run prior to exhaustion was recorded. Exhaustion was defined as the inability of the mouse to maintain running for 30 s.

### CHKB enzyme activity assay in U2O2 cells

U2O2 cells were seeded in T75 flasks one day prior to transfection to achieve ∼90% confluency at the time of transfection. On the day of transfection, the culture medium was replaced with antibiotic-free DMEM containing 10% FBS for 1 h before transfection. A total of 10 μg of plasmid DNA was diluted in 750 μL of Opti-MEM and mixed with 25 μL of Lipofectamine 2000 diluted in 750 μL of Opti-MEM. The mixture was incubated for 30 min at room temperature and then added to each flask. Forty-eight hours after transfection, cells were trypsinized, collected by centrifugation, and pooled (two flasks per experimental group) to generate cell pellets. Pellets were lysed in 200 μL of ice-cold lysis buffer containing 20 mM Tris-HCl (pH 7.5), 145 mM KCl, 2 mM 2-mercaptoethanol, and cOmplete Protease Inhibitor Cocktail (Roche). For every 10 mL of lysis buffer, one tablet of the protease inhibitor was dissolved in 200 μL of 0.1 M phosphate buffer. Lysates were snap-frozen in liquid nitrogen and stored at −80°C until analysis. Protein concentrations were determined using the Bradford assay (calibration range 0.025–2 mg/mL), and samples were diluted 1:20 before measurement. CHKB enzyme activity reactions contained 1,100 μg of total protein (adjusted to a final volume of 300 μL with lysis buffer as needed) and 300 μL of assay buffer (100 mM Tris-HCl, pH 8.75; 10 mM ATP [Na_2_ salt]; and 15 mM MgCl_2_).

### CHKB enzyme activity in mouse tissues

For snap-frozen tissue samples, lysates were prepared using the same lysis buffer. Gastrocnemius muscle was finely minced on ice and homogenized in 10 volumes of buffer using a TissueLyser II (Qiagen) at 30 strokes/s for 3 min with a 1 min cooling interval. Homogenates were centrifuged at 16,000 × *g* for 10 min at 4°C, and the supernatant was collected. Protein concentrations were measured by the Bradford assay (calibration range 0.05–1 mg/mL) and diluted 1:10 before measurement. CHKB activity reactions contained 300 μg of total protein (adjusted to 120 μL with lysis buffer) and 480 μL of assay buffer (100 mM Tris-HCl, pH 8.75; 10 mM ATP [Na_2_ salt]; and 15 mM MgCl_2_).

### LC-MS quantification

Reactions were performed in a total volume of 200 μL in a water bath at 37°C and terminated at time 0 min (baseline phosphocholine) and 20 min by heating at 95°C for 5 min. Samples were centrifuged to pellet precipitated proteins, and supernatants were collected for liquid chromatography-mass spectrometry (LC-MS) analysis. For phosphocholine quantification, 10 μL of each supernatant was spiked with 5 μL of d_9_-phosphocholine internal standard (5 μg/mL in water). Metabolites were extracted with 90 μL of ethanol/methanol/acetonitrile (20:20:60, v/v/v), vortexed briefly, incubated at −20°C for 30 min, vortexed again, incubated at 4°C for 30 min, and centrifuged at 12,000 × *g* for 5 min. The supernatant (80 μL) was used for LC-MS injection. A system suitability control (SSC) was prepared in parallel by substituting water for sample material. A standard curve of d_9_-phosphocholine (10–0.3125 μg/mL) was prepared by serial 2-fold dilution of a 10 μg/mL stock. Targeted LC-MS analysis was performed on a Q Exactive mass spectrometer (Thermo Fisher Scientific) coupled to a Vanquish ultra-high-performance—liquid chromatography (UHPLC) system, controlled by Xcalibur v4.2. Separation was achieved on an Acquity Premier bridged ethyl hybrid (BEH) hydrophobic interaction liquid chromatagraphy (HILIC) column (2.1 × 150 mm, 1.7 μm; Waters) at 0.25 mL/min. The LC gradient began with 95% mobile phase B (5 mM ammonium acetate, 5 mM ammonium hydroxide, and 5% H_2_O in acetonitrile) for 2 min, linearly decreased to 30% B over 0.9 min, then to 0% B in 0.1 min, held for 1 min, and ramped back to 95% B over 0.5 min with equilibration for 4.5 min (total run time = 10 min). Mobile phase A consisted of 5 mM ammonium acetate, 5 mM ammonium hydroxide, and 5% acetonitrile in water. Data were acquired in positive ion mode using a targeted parallel reaction monitoring (PRM) method (resolution 17,500; AGC target 1 × 10^6^; maximum injection time (IT) 50 ms; isolation window 0.7 m/z). Monitored transitions were phosphocholine (precursor m/z 184.07 → fragment m/z 60) and d_9_-phosphocholine (precursor m/z 193.12 → fragment m/z 60) using NCE 30. Additional parameters were as follows: sheath gas 45, auxiliary gas 20, sweep gas 2, spray voltage 3.7 kV, capillary temperature 300°C, S-lens RF 45, and auxiliary gas heater 400°C. Raw data were processed in Skyline. CHKB kinase activity was calculated from the net increase in phosphocholine between 0 min and 20 min, normalized to d_9_-phosphocholine. Enzyme activity was expressed as normalized peak area change per min per mL of protein extract.

### Measurement of AST and ALT enzyme activity

Hepatic AST and ALT activities were measured in plasma obtained from blood samples collected via cheek bleed using RAM Scientific Safe-T-Fill Capillary Blood Collection Systems: EDTA (Cat. No. 077051; RAM Scientific, USA). Blood was centrifuged at 3,000 × *g* for 10 min at 4°C to separate plasma. Twenty microliters (20 μL) of plasma was used for analysis with the Aspartate Aminotransferase Activity Assay Kit (Cat. No. 701640) and the Alanine Transaminase Activity Assay Kit (Cat. No. 700260; Cayman Chemical, Ann Arbor, MI, USA), following the manufacturer’s instructions.

### Preparation of frozen tissue sections for subsequent H+E and immunofluorescence staining

Following dissection, collected tissues were weighed, mounted on cue cards using Optimal Cutting Temperature (OCT) compound (Sakura Finetek, Torrance, CA, USA), and frozen in liquid-nitrogen-cooled isopentane. Samples were stored at −80°C until further processing. Frozen sections (10 μm thick) were cut and thaw-mounted onto SuperFrost microscope slides (Microm International, Kalamazoo, MI, USA) and air-dried at room temperature. Slides were stored at −20°C until subsequent hematoxylin and eosin (H+E) or IF staining.

### Tissue histology characterization using H+E

Frozen tissue sections were stained using a Leica Spectra automated stainer following a modified SelecTech protocol. Slides were first washed in distilled water for 1 min then stained in SelecTech Hematoxylin 560 MX (Leica Biosystems; Cat. No. 3801575 or 3801576) for 2–3 min, depending on section thickness. Slides were rinsed in distilled water for 2 min and differentiated in SelecTech Define (20× stock; Leica Biosystems; Cat. No. 3803596 or 3803595) for 1 min, followed by a 1 min water rinse. Bluing was performed with SelecTech Blue (20× stock; Leica Biosystems; Cat. No. 3802916 or 3802915) for 1 min followed by an additional water rinse. Sections were then dehydrated through graded ethanol solutions, starting with 95% ethanol for 1 min, counterstained with SelecTech Eosin 515LT (Leica Biosystems; Cat. No. 3801619) for 3–5 s, and further dehydrated in 70% ethanol for 1.5 min, 95% ethanol for 1 min, and two changes of 100% ethanol for 1 min each. Clearing was performed in two changes of xylene for 1 min each, followed by a brief 1 s transfer through the final station before cover slipping.

### CHKB immunofluorescence of tissue sections

For CHKB IF, frozen muscle sections were fixed in cold methanol for 7 min at −20°C, followed by three washes in PBS for 5 min each. Sections were blocked for 30 min at room temperature using SuperBlock (PBS) Blocking Buffer (Thermo Scientific), then incubated overnight at 4°C with anti-CHKB antibody (sc-398957; Santa Cruz Biotechnology, Dallas, TX, USA) diluted 1:20 in 1% bovine serum albumin (BSA) in PBS. After incubation, sections were washed three times in PBS (5 min each) and incubated for 1 h at room temperature with *m*-IgGκ BP-CFL 594 secondary antibody (sc-516178; Santa Cruz Biotechnology) diluted 1:100 in 1% BSA in PBS. Sections were washed three additional times in PBS (10 min each). After staining, all sections were gently tap-dried and mounted using ProLong Diamond Antifade Mountant with DAPI (Thermo Scientific, Cat. No. P36966). Slides were allowed to cure overnight in the dark before imaging. Fluorescence images were acquired using a Zeiss Axio Observer Z.1 Spinning Disk Confocal Microscope operated with ZEN Black software (Zeiss, Oberkochen, Germany).

### Measurement of triacylglycerol amount

Snap-frozen tissue samples were homogenized in 4 volumes of lysis buffer on ice. Ten microliters (10 μL) of the homogenate were used to quantify tissue triglyceride levels using the EnzyChrom Triglyceride Assay Kit (BioAssay Systems, Cat. No. ETGA-200), following the manufacturer’s instructions. Measurements were performed in technical duplicates, and values were normalized to total protein content determined by the Bradford assay.

### Total RNA isolation, cDNA generation, and quantitative real-time qPCR

Flash-frozen tissue samples were homogenized in TRIzol Reagent (Invitrogen, Carlsbad, CA, USA; Cat. No. 15596026) using a TissueLyser II (Qiagen, Hilden, Germany) at 30 strokes/sec for 3 min, with a 1 min cooling interval to prevent overheating. Total RNA was isolated according to the manufacturer’s instructions. A total of 1.1 μg of RNA was reverse-transcribed using the High-Capacity cDNA Reverse Transcription Kit (Applied Biosystems, Foster City, CA, USA; Cat. No. 4368814). Quantitative real-time PCR (qPCR) was performed on a Bio-Rad CFX Duet Real-Time PCR Detection System (Bio-Rad, Hercules, CA, USA; Part No. 12016265) using TaqMan Fast Advanced Master Mix (Thermo Fisher Scientific, Waltham, MA, USA; Cat. No. 4444557) and TaqMan Gene Expression Assays (Thermo Fisher Scientific; Cat. No. 4331182) for the following targets: Cpt1b (RRID: Mm00487191_g1), Nd1 (RRID: Mm04225274_s1), Icam1 (RRID: Mm00516023_m1), Tgfb1 (RRID: Mm01178820_m1), Tnfa (RRID: Mm00443258_m1), and Rplp0 (RRID: Mm00725448_s1). All reactions were performed in technical triplicates. Data acquisition and analysis were conducted using CFX Maestro Software v.2.3 (Bio-Rad). Relative gene expression was calculated using the ΔΔCt method with Rplp0 as the endogenous control.

### Western blot analysis and quantification

Tissue samples were lysed, and total protein content was quantified as described in the determination of CHKB enzyme activity by LC-MS. Based on protein quantification results, all samples were adjusted to equal concentrations and heat-denatured for 5 min at 99°C in 2× Laemmli buffer. A total of 45 μg of protein from skeletal muscle, 70 μg from cardiac muscle, or 6 μg from CHKB-overexpressing U2OS cells were separated by SDS-PAGE and transferred onto nitrocellulose membranes. Transfer efficiency was confirmed by Ponceau S staining. Membranes were blocked for 1 h at room temperature in SuperBlock (Thermo Scientific) for CHKB detection or Odyssey Blocking Buffer (LI-COR Biosciences) for other targets. Membranes were incubated overnight at 4°C with the following primary antibodies: Anti-CHKB (1:250; Santa Cruz Biotechnology, Cat. No. sc-398957), Anti-CPT1 (1:1000; Proteintech, Cat. No. 22170-1-AP), Anti-GAPDH (1:1000; Cell Signaling Technology, Cat. No. 2118). After washing, membranes were incubated for 1 h at room temperature with either goat anti-rabbit IRDye 800CW secondary antibody (1:20,000; LI-COR Biosciences, Cat. No. 926–32211) or anti-mouse *m*-IgGκ BP–CFL 790 secondary antibody (1:20,000; Santa Cruz Biotechnology, Cat. No. sc-516181). Protein bands were visualized using an Odyssey Imaging System (LI-COR Biosciences) and band intensities quantified using FIJI software.

### Statistical analysis

Statistical analyses were performed using GraphPad Prism (v.10.2). Data were analyzed using an unpaired *t* test, or an ordinary one-way analysis of variance (ANOVA) without matching or pairing, as required. Data were assumed to follow a Gaussian (normal) distribution with homogeneity of variance across groups. When the ANOVA indicated a significant overall group effect, Dunnett’s multiple-comparisons test was used to compare each experimental group. Data are presented as mean ± standard deviation (SD). Statistical significance was defined as *p* < 0.05.

## Data and code availability

Data supporting the findings of this study are present in the manuscript and are available from the corresponding author.

## Acknowledgments

The work was supported by a grant from the 10.13039/501100000024Canadian Institutes of Health Research (SOP-159230) to C.R.M.

## Author contributions

M.T., conceptualization, investigation, methodology, supervision, data curation, formal analysis, validation, visualization, writing – original draft, and writing – review and editing; M.A., investigation, methodology, data curation, formal analysis, validation, and writing – review and editing; G.D., investigation, data curation, and writing – review and editing; L.H., conceptualization, methodology, validation, and writing – review and editing; J. Devitt, investigation, methodology; J. Damsker, conceptualization and writing – review and editing; E.P.H., conceptualization, validation, supervision, writing – original draft, and writing – review and editing; and C.R.M., conceptualization, supervision, project administration, validation, writing – original draft, writing – review and editing, and funding acquisition.

## Declaration of interests

The authors report no competing interests.

## Supplemental information

Document S1. Figures S1–S4

Document S2. Article plus supplemental information
