# Supplementary material for: Preclinical efficacy of a gene therapy for CHKB-mediated muscular dystrophy
Source: Mol Ther Adv. 2026 May 25;34(3):201766. doi: 10.1016/j.omta.2026.201766 (PMC13263696; doi:10.1016/j.omta.2026.201766)
Supplement: Document S1. Figures S1–S4 [file mmc1.pdf]

OMTA, Volume *34*

Supplemental information

Preclinical efficacy of a gene therapy for *CHKB*-mediated muscular dystrophy

Mahtab Tavasoli, Mariam Alkandari, Gabriel Dorighello, Jennifer Devitt, Laura Hagerty, Jesse Damsker, Eric P. Hoffman, and Christopher R. McMaster

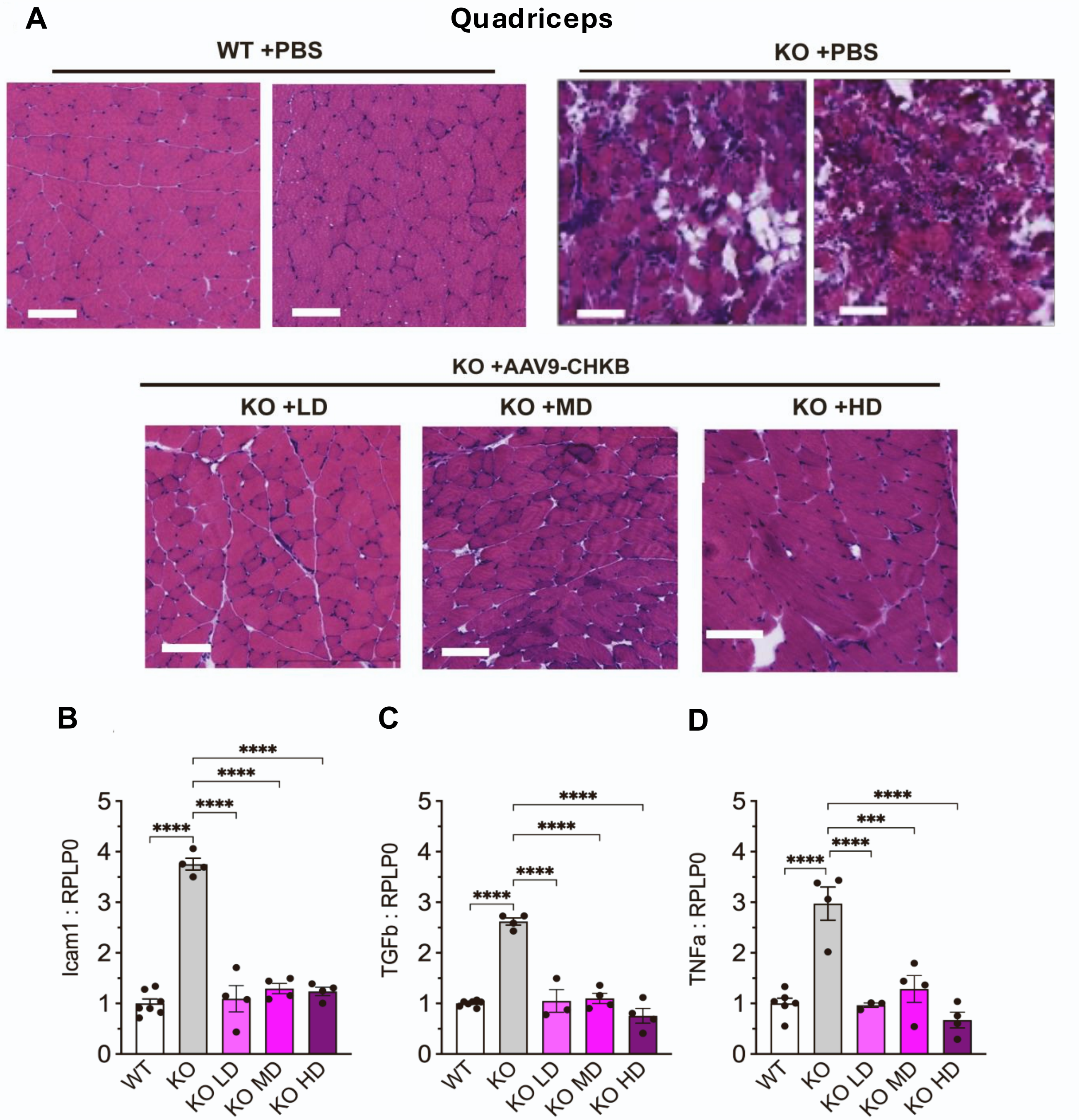

Figure S1. Histopathology of quadriceps and triceps muscles in *Chkb*$^{-/-}$ mice. (A) H+E-stained quadriceps muscle sections from WT and *Chkb*$^{-/-}$ mice, and *Chkb*$^{-/-}$ mice treated with low (LD, 5 x 10^{13} vg/kg), medium (MD, 1 x 10^{14} vg/kg), or high (HD, 2 x 10^{14} vg/kg) doses of AAV9-*CHKB*. Quadriceps from *Chkb*$^{-/-}$ animals show dystrophic features similar to those observed in gastrocnemius (Fig. 5), including fiber size variability and necrosis. AAV9-*CHKB* treatment restored quadriceps histology to WT morphology, with minimal differences between doses. Scale bars, 100 µm. **(B-D)** Expression of muscle injury larkers in quadriceps in WT and *Chkb*$^{-/-}$ mice, and *Chkb*$^{-/-}$ mice treated with AAV9-*CHKB* as determined by RT qPCR. All three muscle injury markers increased from 2.5-fold to 4.0-fold in *Chkb*$^{-/-}$ mice compared to wild type with the level of all markers restored to WT by AAV9-*CHKB* treatment.

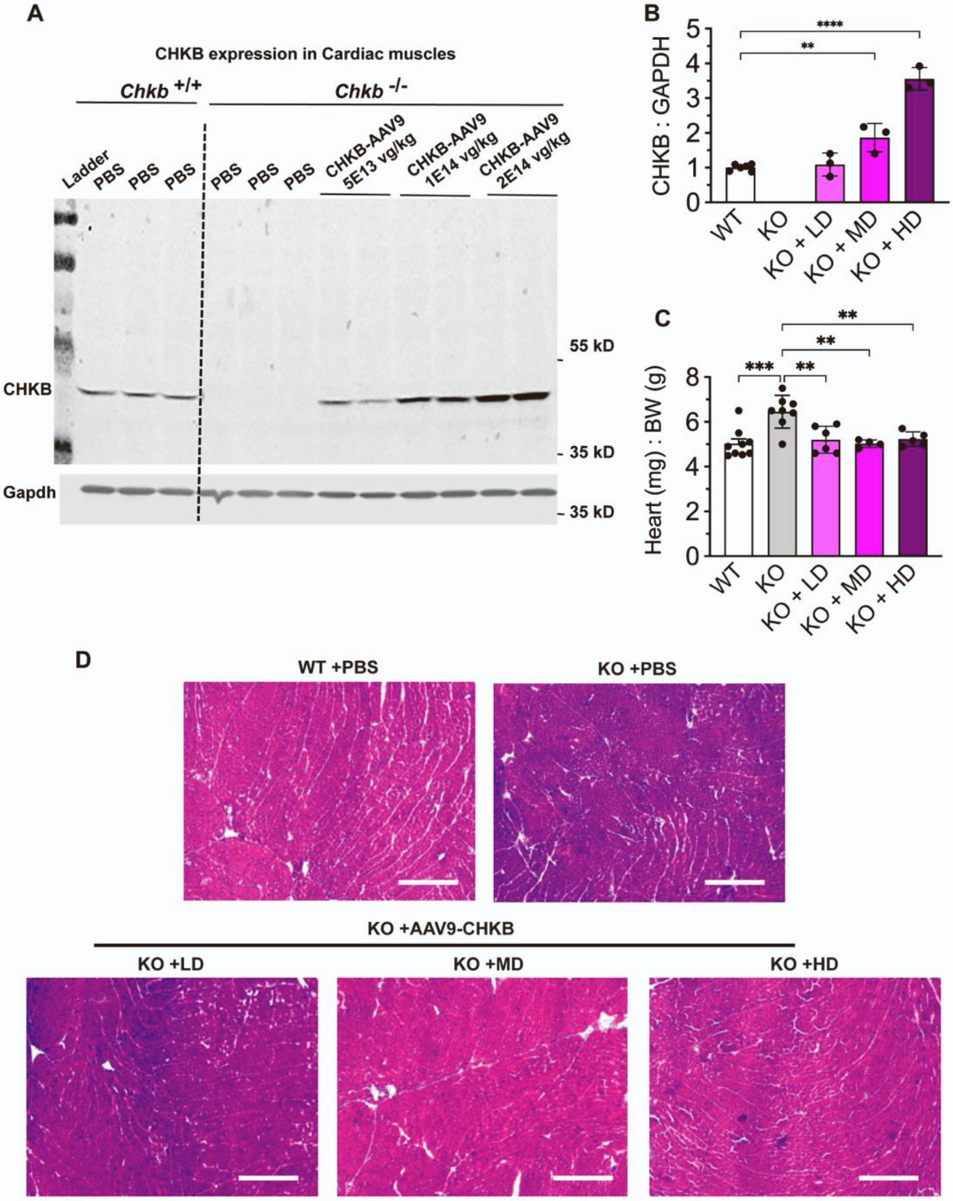

Figure S2. Cardiac phenotypes are normalized by AAV-*CHKB* treatment. (A) Western blot of CHKB protein in cardiac tissue from WT, KO, and KO + AAV9-CHKB mice (doses: 5 × 10^13, 1 × 10^14, 2 × 10^14 vg/kg) probed with anti-CHKB, and anti-Gapdh antibodies. (**B**) Heart weight normalized to body weight (mg/g) shows significant cardiac hypertrophy in untreated KO mice which is corrected after AAV9-CHKB treatment. n = 9 (WT), n = 8 (KO), n = 6 (KO+LD), n = 4 (KO+MD) and n = 5 (KO+HD) mice per group. One-way ANOVA with Tukey's multiple comparison test. Data are mean ± SD; dots indicate individual mouse. **P < 0.01, ***P < 0.001. (**C**) H&E-stained cardiac sections demonstrate similar overall morphology across WT, KO, and AAV9-CHHKB-treated KO hearts, indicating no overt histological abnormalities between groups. Scale bars=100 uM. Data are mean +/- SD; individual replicates are shown as dots. **P<0.01, ***P<0.001. Scale bars, 100 µm.

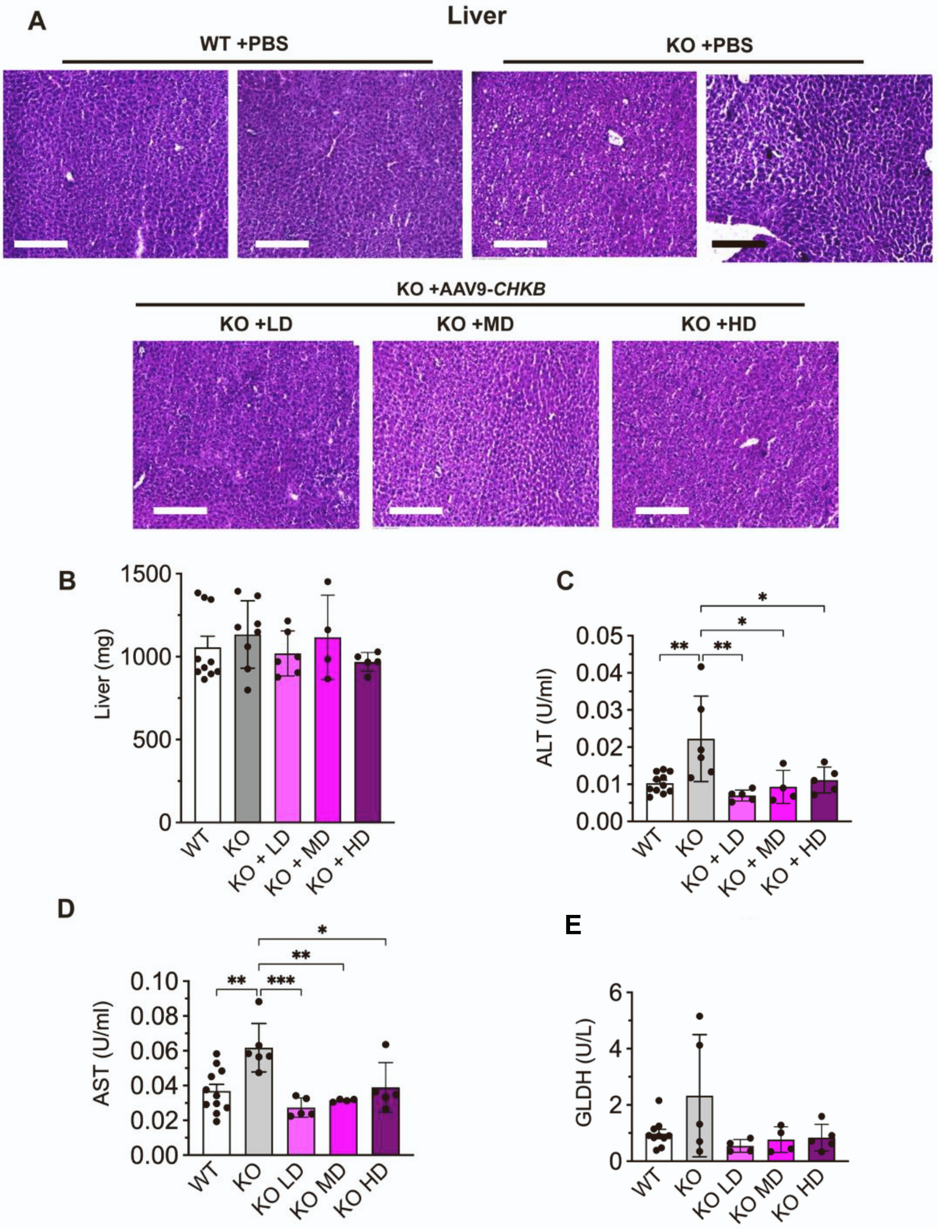

Figure S3. Liver histology and serum biomarkers demonstrate preserved hepatic integrity following systemic AAV9-*CHKB* delivery. (A) Representative H+E-stained liver sections from WT and *Chkb*$^{-/-}$ mice, as well as and *Chkb*$^{-/-}$ mice treated with low (LD, 5 x 10^{13} vg/kg), medium (MD, 1 x 10^{14} vg/kg), or high (HD, 2 x 10^{14} vg/kg) doses of AAV9-*CHKB*. All groups, including untreated KO, show preserved lobular architecture without evidence of necrosis, fibrosis, or fatty infiltration. Scale bars, 100 µm. **(B)** Liver weight was determined in WT and *Chkb*$^{-/-}$ mice, as well as and *Chkb*$^{-/-}$ mice treated with LD, MD, or HD dose AAV9-*CHKB*. **(C,D)** AST and ALT activities are elevated 2-fold in *Chkb*$^{-/-}$ mice compared with WT and restored to WT level by AAV9-*CHKB* treatment. **(E)** The FDA approved marker of liver cell damage, GLDH, was also elevated 2-fold in *Chkb*$^{-/-}$ mice compared with WT and restored to WT level by AAV9-*CHKB* treatment. Data are presented as mean ± SD; dots indicate individual mice. One-way ANOVA with Tukey's multiple comparison test. $^{*}P < 0.05$, $^{**}P < 0.01$, $^{***}P < 0.001$, $^{****}P < 0.0001$.

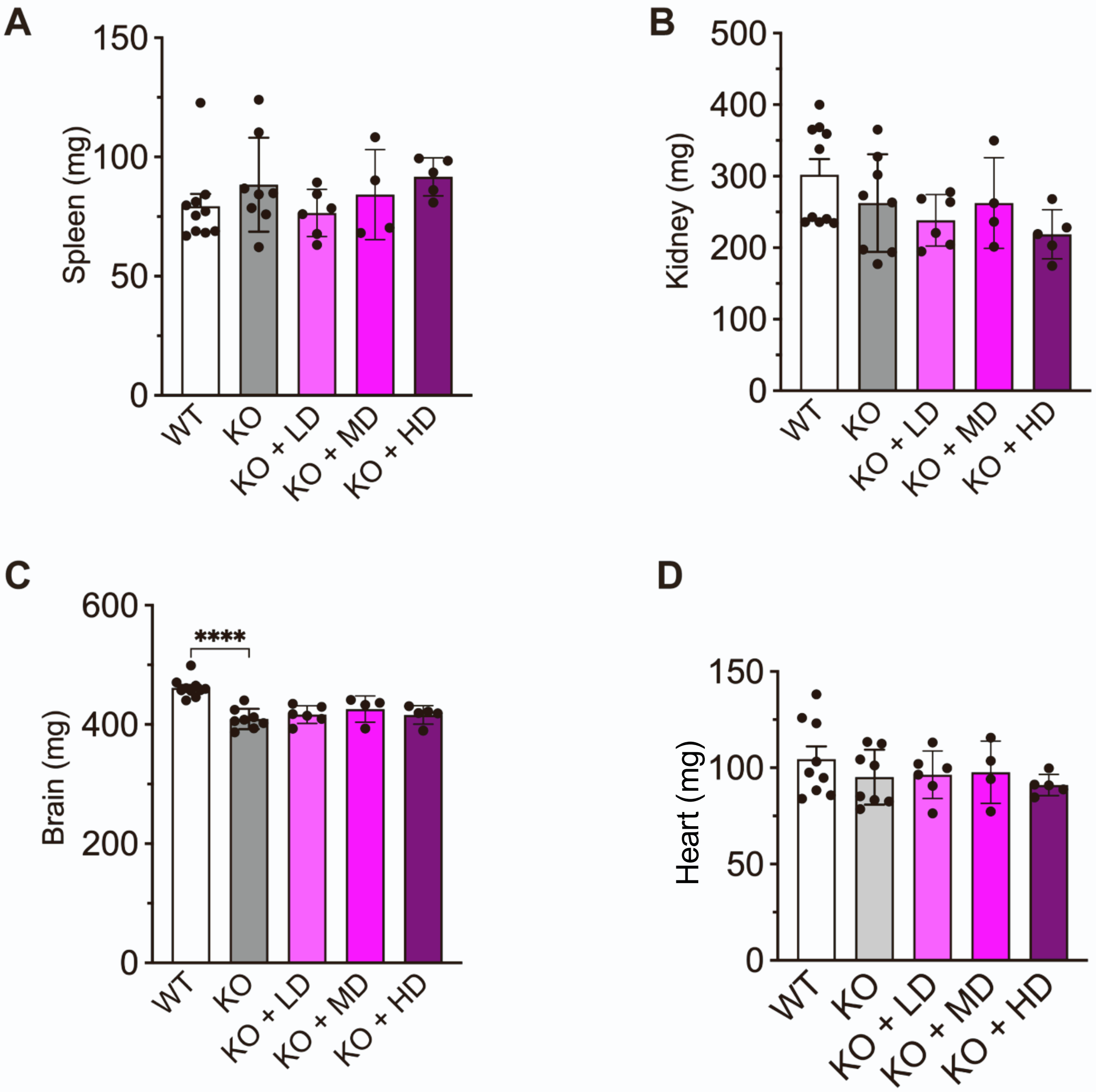

Figure S4. Organ weights in WT, *Chkb*$^{-/-}$ and *Chkb*$^{-/-}$ AAV9-CHKB treated mice. Organ weights of (A) spleen, (B) kidney), (C) brain, and (D) heart of WT, *Chklb*$^{-/-}$ (KO) and *Chkb*$^{-/-}$,ice treated with low (LD, 5 x10^{13} vg/kg) medium (MD, 1 x 10^{14} vg/kg, and and high (HD, 2 x 10^{14} vg/kg). Data are the mean + SD. Dots represent individual mice. Statistical analysis was performed using a one-way ANOVA with Tukey's multiple comparison test, **** $P < 0.0001$.
